# MEMORY IN LATER LIFE: HOW NONAGENARIANS AVOID FORGETTING WHAT THEY WANT TO REMEMBER

**DOI:** 10.1093/geroni/igae098.2655

**Published:** 2024-12-31

**Authors:** Katie Cherry, Matthew Willis, Gavin Faucheux, Dominique Bernard, Mary Barnes, Celinda Reese-Melancon, Sangkyu Kim, S Michal Jazwinski

**Affiliations:** Louisiana State University, Baton Rouge, Louisiana, United States; Louisiana State University, Baton Rouge, Louisiana, United States; Louisiana State University, Baton Rouge, Louisiana, United States; Louisiana State University, Baton Rouge, Louisiana, United States; Louisiana State University, Baton Rouge, Louisiana, United States; Oklahoma State University, Stillwater, Oklahoma, United States; Tulane University School of Medicine, New Orleans, Louisiana, United States; Tulane University School of Medicine, New Orleans, Louisiana, United States

## Abstract

Subjective perceptions of memory play an important role in cognitive adaptability in later life. While self-reported memory is typically assessed using objective surveys, few studies have examined what older people have to say about their own experiences using memory in everyday life. The purpose of this study was to examine older adults’ memory management strategies and techniques that they use in everyday life to improve recall of information they do not want to forget. Participants consisted of 50 nonagenarians (90+ years of age) and two younger reference groups (60-74 and 75-89 years) who were enrolled in the Louisiana Healthy Aging Study (LHAS). All answered two open-ended questions regarding their preferred memory management strategies used in everyday life. Narrative responses were recorded, transcribed verbatim, and content analyzed for recurring concepts. Results yielded new evidence of environmental strategies, including object placement and non-electronic external memory aids (e.g., planners, calendars), and internal mnemonic techniques (e.g., rehearsal, association). When comparing internal mnemonic techniques specifically, nonagenarians were more likely to use association, whereas their younger counterparts were more likely to use imagery. These findings suggest that memory management techniques change very little in late adulthood. This research was supported grants from by the Louisiana Board of Regents through the Millennium Trust Health Excellence Fund [HEF(2001-06)-02] and the National Institute on Aging (P01 AG022064).

